# Pupillary response to dynamic pitch alteration during speech perception in noise

**DOI:** 10.1121/10.0007056

**Published:** 2021-11-05

**Authors:** Jing Shen

**Affiliations:** Department of Communication Sciences and Disorders, Temple University, 1701 North 13th Street, Philadelphia, Pennsylvania 19122, USA jing.shen@temple.edu

## Abstract

Dynamic pitch, also known as intonation, conveys both semantic and pragmatic meaning in speech communication. While alteration of this cue is detrimental to speech intelligibility in noise, the mechanism involved is poorly understood. Using the psychophysiological measure of task-evoked pupillary response, this study examined the perceptual effect of altered dynamic pitch cues on speech perception in noise. The data showed that pupil dilation increased with dynamic pitch strength in a sentence recognition in noise task. Taken together with recognition accuracy data, the results suggest the involvement of perceptual arousal in speech perception with dynamic pitch alteration.

## 1. Introduction

Dynamic pitch, as defined by the variation in fundamental frequency of speech, is one of the major acoustic cues in speech. Also known as intonation, dynamic pitch conveys both semantic and pragmatic meaning in communication.^1,2^ The research evidence on the role of dynamic pitch in speech perception comes from multiple fields. Data from hearing science has shown that speech is less intelligible in background noise when natural dynamic pitch cues are flattened or altered.^3–5^ This effect is also modulated by acoustic factors including noise type^6^ and amount of alteration.^7^ While these findings are based on speech intelligibility measures of speech reception thresholds^3,7^ or speech recognition accuracy,^5,8^ these measures may not provide a complete picture of the role of dynamic pitch in speech perception in noise. Support for this argument comes from psycholinguistics and communication research, which has demonstrated the role of dynamic pitch in speech processing with a wider range of dependent measures. For instance, Braun and colleagues altered the pitch contour of Dutch sentences by replacing it with a sinusoidal pattern, either by resynthesis or natural production.^9^ Their data demonstrated a detrimental effect of the altered intonation on processing speed and accuracy across multiple tasks including word monitoring, lexical decision, and semantic categorization. Using stimuli of radio commercial messages, Rodero *et al.*^10^ examined listeners' responses with different overall pitch and variation patterns. The dependent variables included subjective measures of perceived effectiveness and adequacy, objective measures of immediate recall, and psychophysiological measures of arousal and attention, as indicated by skin conductance level and heart rate. The results showed a consistent effect of dynamic pitch patterns across multiple dependent measures. The pattern with a larger pitch range and higher pitch in the first half of the segment and lower pitch in the latter half of the segment (i.e., H-L pitch pattern) elicited higher scores and better performances across all the measures. It is worth noting that there was a significant effect of pitch pattern on the arousal measure of skin conductance level. The overall skin conductance level was higher and had a more sustained increase in the H-L pitch condition than in all other conditions. Along the same line, event-related potentials (ERPs) data showed that emotional prosody was processed in a covert (i.e., non-voluntary) manner.^11^ In a lexical decision task that was designed to direct listeners' attention away from prosody, the ERP data showed early and non-voluntary processing of prosodic valence. This finding suggests prosodic cues such as dynamic pitch can influence physiological measures even when listeners were not engaging in active processing of prosodic information. Built on these findings, it is hypothesized that the effect of dynamic pitch cue is more than just intelligibility and may include other aspects of speech processing, such as arousal and covert attention. One psychophysiological measure that has been used by research on arousal and attention is task-evoked pupillary response,^12–14^ which takes pupil size as an indication of changes in arousal level induced by specific stimuli/tasks.

It is important to note that the measure of task-evoked pupil response can also provide information on the processing effort associated with speech perception under adverse conditions.^15^ Research in this area has provided evidence showing a large pupillary response with speech perception tasks with strong signal degradation,^16,17^ high linguistic complexity, and a high level of processing.^18,19^ Based on the intelligibility data, it is reasonable to expect that altered dynamic pitch cue makes speech recognition in noise more effortful due to alteration of the original acoustic cues. On the other hand, it is also possible that the effect of altered dynamic pitch on speech perception involves more than one mechanism of effortful processing of speech signal in noise. Built upon the previous studies,^10,11^ it is hypothesized that altered dynamic pitch can make speech sound more exciting and induce changes in arousal level during speech perception, which can only be demonstrated by pupil response but not intelligibility measure. Following this rationale, we measured task-evoked pupillary responses with altered dynamic pitch cues when speech was perceived in noise. If the altered dynamic pitch conditions are more difficult to be perceived, we would expect intelligibility data to align with previous findings,^7^ along with a consistent pattern in pupillary response indicating processing effort (i.e., larger processing effort with weakened/strengthened dynamic pitch as compared to original one). The pupil response data, however, could also be influenced by perceptual arousal due to dynamic pitch cues and show a more complex pattern.

## 2. Methods

### 2.1 Participants

Twenty young participants (mean age: 22.5 years, range: 19–31 years) with normal hearing (Pure Tone Average < 20 dB HL) participated in this experiment. Nineteen participants self-identified as female and one as other. One participant's data were removed from analysis due to technical errors. All participants were native speakers of General American English. They were recruited from the Temple University community and paid for their time. The study protocol was approved by the Institutional Review Board of Temple University.

### 2.2 Stimuli

Stimuli were taken from the Institute of Electrical and Electronics Engineers (IEEE)/Harvard sentence corpus,^20^ with five keywords in each sentence. To avoid potential confounds from linguistic and affective outliers on arousal level, the IEEE sentences were reviewed by two native speakers of English to remove sentences that may increase listeners' arousal level (e.g., “The prince ordered his head to be chopped off”). The stimuli were produced by a female native speaker of General American English. Following the same alteration strategies used in previous research,^3,7^ the sentences were resynthesized to have altered dynamic pitch cues using the Praat program.^21^ Three dynamic pitch conditions include original dynamic pitch (by keeping the original pitch contour but processed using the same resynthesis method), strengthened dynamic pitch (1.4 times original pitch contour), and weakened dynamic pitch (0.5 times original pitch contour). The mean duration of all the sentences was 2668 ms with a range from 1946 to 3292 ms. The sentences were embedded in non-speech noise that preserved the temporal and spectral characteristics of 2-talker babble [International Collegium of Rehabilitative Audiology (ICRA) 2-talker noise].^22^ Two conditions with signal-to-noise-ratio of –5 dB signal-to-noise ratio (SNR) and –9 dB SNR were included based on piloting data collected from six participants who did not participate in this study. These two noise conditions were used to keep the speech recognition accuracy in the range of 70%–90% correctly recognized keywords. There were six conditions in total (3 pitch conditions × 2 noise conditions) and 2 lists of 13 sentences were presented for each condition.

### 2.3 Procedure

The participant was seated in a dimly lit double-walled sound booth^23^ in front of a BenQ Zowie liquid crystal display (LCD) monitor. The distance between eye and screen was 60 cm. The pupil diameter data were collected using an Eyelink 1000 plus eye-tracker in remote mode with head support. The sampling rate was 1000 Hz, and the participants' left eye was tracked. Following methods used in previous research,^24^ the color of the screen was set to be {120 120 120} in red-green-blue (RGB) color code based on piloting to avoid limits of the range of pupil size. The luminance measure was 37 lux at the eye position.

The experiment was implemented with a customized program using Eyelink Toolbox^25^ in matlab. Auditory stimuli were presented over a Sennheiser HD-25 headphone at 65 dBA. For each trial, a red cross (RGB {220 30 100}) was first presented on the center of the screen. The participant was instructed to look at the sign once it appeared. After 2000 ms of silence (baseline period), the audio stimuli were played. Each stimulus started with background noise and the sentences were time-aligned based on the offset. The sentence began about 2000 ms after the noise onset, depending on the sentence length. The total length of the audio stimuli was 4500 ms. There was a retention period of 2000 ms after the stimuli finished playing. After that, the red cross-sign was replaced by a green sign (RGB {30 140 30}) and the participant was instructed to provide a verbal response by repeating back what they heard only after the green sign appears. Once the participant finished talking, the tester terminated the trial with a key press and a gray box (RGB {75 75 75}) appeared on the screen center after 1000 ms of silence. The participant then had a brief resting period for 5000 ms, before the next trial started.

Prior to testing, the participant had a brief practice session consisting of six trials (with two trials for each pitch condition, ourf trials in –5 dB SNR condition, and two trials in –9 dB SNR condition) to familiarize them with the procedure. The testing included 12 blocks of 13 trials each, with condition order counterbalanced by Latin Square Design. The experiment took 90 min in total and the participants were given a break every four blocks to reduce fatigue.

## 3. Results

**Fig. 1.**
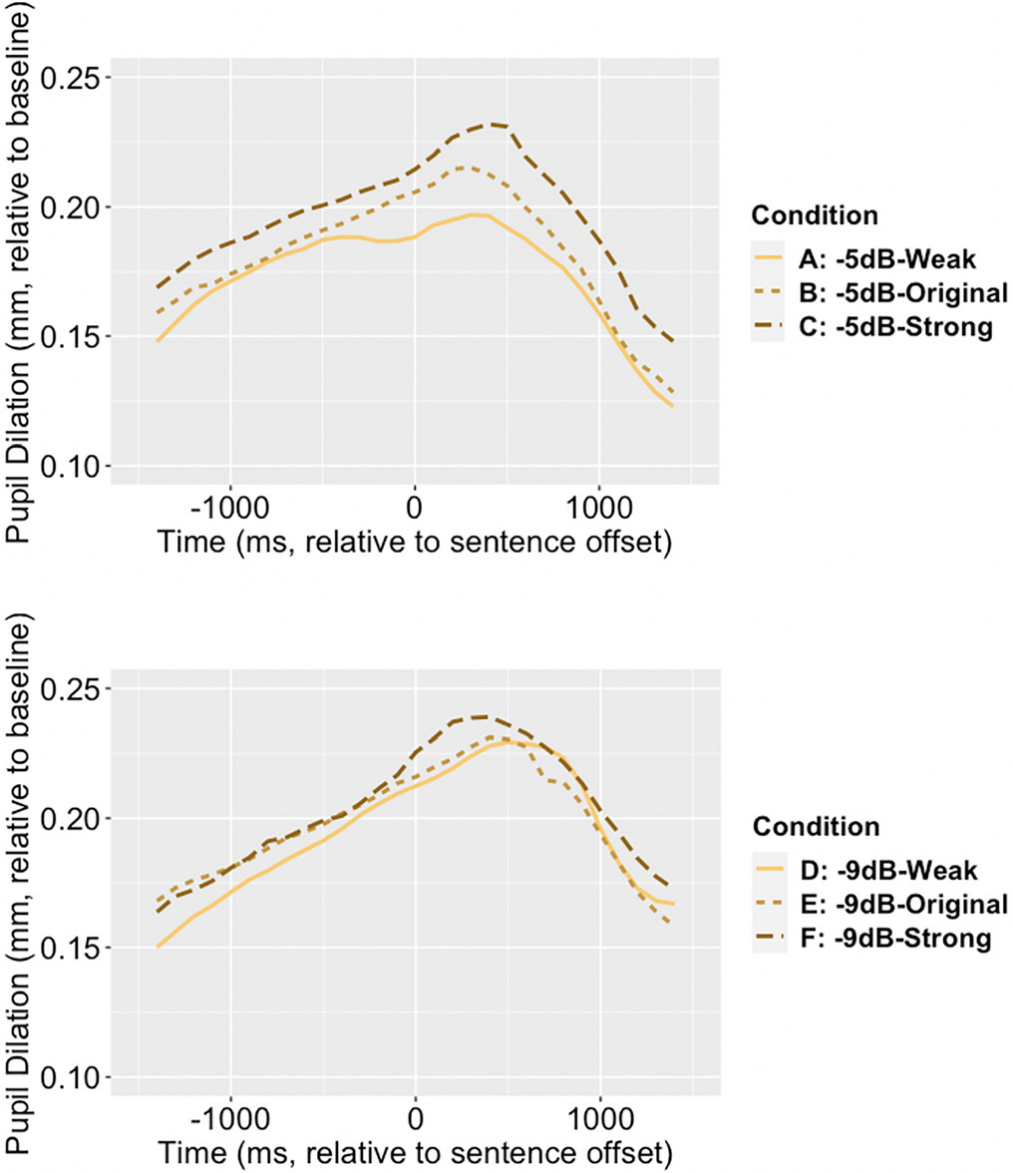
Pupil dilation (mm) as a function of time. Top panel, favorable noise (−5 dB SNR); bottom panel, adverse noise (−9 dB SNR).

Pupil diameter data were pre-processed using R (Version 3.2.1) with GazeR library^26^ and down sampled to 10 Hz before analysis. De-blinking was implemented between 100 ms before the blink and 100 ms after the blink. The curve was linearly interpolated and smoothed using a 20-point moving average. The dependent measure was pupil diameter relative to individuals' baseline level of each trial (recorded at the first 2000 ms silent period). Figure 1 shows pupil dilation (i.e., the pupil size relative to baseline) during the time window from −1500 to +1500 ms relative to sentence offset, collapsed across participants.

Growth curve analysis^27^ was used to analyze pupil diameter data within the time window from −1500 to 500 ms relative to sentence offset. This window was chosen based on the lower limit of the sentence duration to avoid inclusion of response to background noise preceding the sentence. Data were first analyzed with one model to confirm the effect of noise [*χ*^2^ (1) = 87.454, *p* < 0.001]. After that, the data in two noise conditions were analyzed with two separate models to facilitate interpretation. The overall pupil dilations were modeled with second-order orthogonal polynomials and fixed effects of pitch conditions on all time terms. The original dynamic pitch condition was treated as the baseline and parameters were estimated for the strengthened and weakened dynamic pitch conditions. The fixed effects of pitch conditions were added individually and their effects on model fit were evaluated using model comparisons. The models also included random effects of participants on all three components of the time course. All analyses were carried out in R version 4.0.3 using the lme4^28^ and lmerTest^29^ packages.

In the more favorable noise condition (−5 dB SNR), the dynamic pitch alteration improved model fit on intercept term [*χ*^2^(2) = 21.947, *p* < 0.001], linear term [*χ*^2^(2) = 33.461, *p* < 0.001] and quadratic term [*χ*^2^(2) = 40.454, *p* < 0.001]. Table 1 shows the fixed effect parameter estimates and their standard errors along with *p*-values using the Satterthwaite approximations to degrees of freedom. Similar results were observed in the more adverse noise condition (−9 dB SNR), with the dynamic pitch alteration improved model fit on intercept term [*χ*^2^(2) = 22.105, *p* < 0.001], the linear term [*χ*^2^(2) =28.059, *p* < 0.001] and quadratic term [*χ*^2^(2) = 27.689, *p* < 0.001]. The parameter estimates are also reported in Table 1.

**Table 1.** Parameter estimates for analysis of dynamic pitch (DP) on pupil size.

	Favorable Noise (−5 dB SNR)				Adverse Noise (−9 dB SNR)			
	Estimate	Std. Error	*t*	*p*	Estimate	Std. Error	*t*	*p*
Intercept	382.96	70.25	5.451	<0.001	360.10	53.12	6.779	<0.001
Linear	−128.17	133.27	−0.962	>0.1	−308.78	124.10	−2.488	<0.05
Quadratic	119.86	91.85	1.305	>0.1	−363.01	129.40	−2.805	<0.05
Strong DP: Intercept	66.66	10.61	6.280	<0.001	46.42	11.14	4.169	<0.001
Strong DP: Linear	−50.22	44.13	−1.138	>0.1	61.44	43.24	1.421	>0.1
Strong DP: Quadratic	206.92	41.54	4.981	<0.001	195.98	46.29	4.234	<0.001
Weak DP: Intercept	42.24	10.55	4.004	<0.001	40.60	11.21	3.622	<0.001
Weak DP: Linear	180.35	39.56	4.559	<0.001	219.40	43.40	5.055	<0.001
Weak DP: Quadratic	228.55	38.32	5.965	<0.001	−33.06	42.14	−0.785	>0.1

**Fig. 2.**
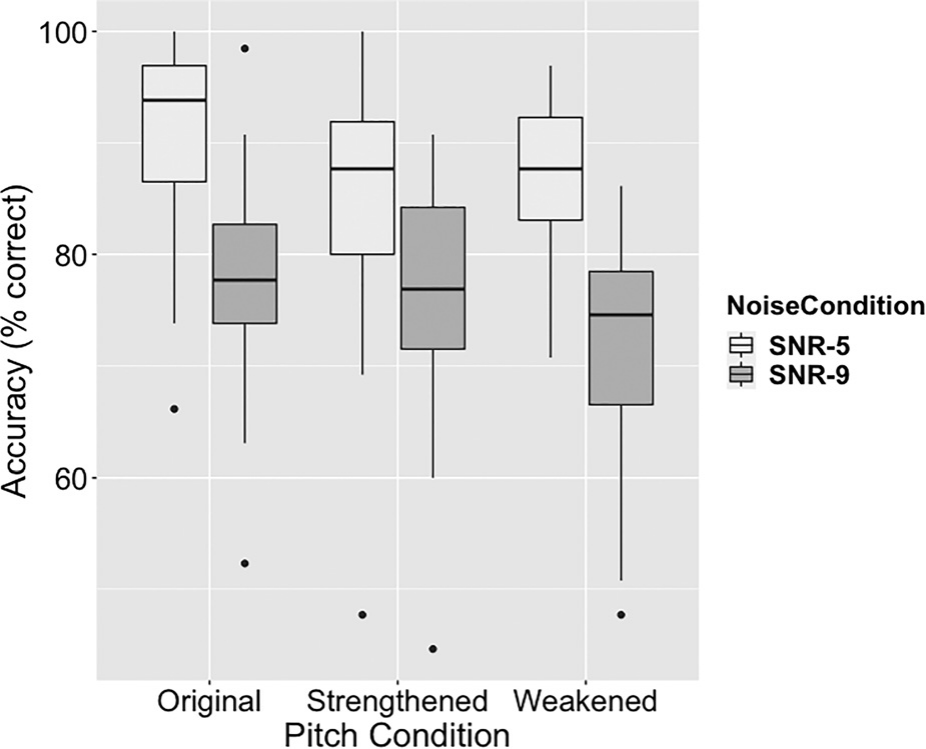
Boxplot of speech recognition accuracy (keyword percent correct) in all dynamic pitch and noise conditions.

To test whether a dynamic pitch and noise conditions predicted recognition accuracy in each of the two noise conditions, speech recognition accuracy (i.e., percent key words correctly recognized) was analyzed using mixed-effects linear regression models.^28^ The dependent variable was speech recognition accuracy (percent key words correct) in each condition. The model included fixed effects for dynamic pitch conditions, order of measurement, and random intercepts for the participant. Simple coding, a coding scheme used in mixed-effects linear regression models to set up contrast in the model, was used to compare the recognition accuracy in the two altered pitch conditions to that in the original pitch condition (which was treated as reference level). The strengthened dynamic pitch had lower accuracy in the more favorable noise condition [*β*= –2.398, *t*(95) = –2.391, *p* < 0.05] and the weakened dynamic pitch had lower accuracy in the more adverse noise condition [*β* = –3.087, *t*(95) = –3.894, *p* < 0.01]. Figure 2 shows the recognition accuracy data in each of the six conditions.

**Fig. 3.**
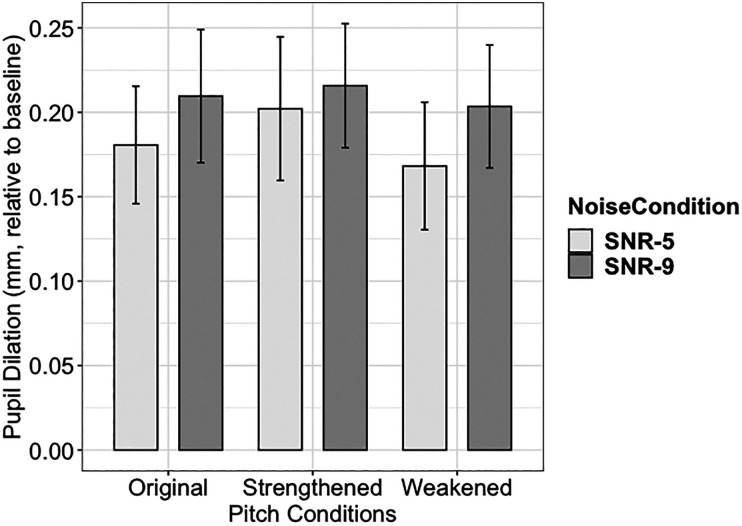
Peak pupil dilation (mm) as relative to baseline in all six conditions. Error bars present standard error.

To examine the question of whether speech recognition accuracy predicted pupil dilation after pitch and noise conditions were accounted for, another analysis was carried out using mixed-effects linear regression models.^28^ The dependent variable was peak pupil dilation (within the window of −500 to 1500 ms relative to sentence offset) relative to baseline level for each trial. The model included fixed effects for dynamic pitch condition, noise condition, recognition accuracy of each block, order of measurement, and random intercepts for participant. Simple coding was used to compare the pupil size in the two altered pitch conditions as compared to that in the original pitch condition. Model comparison showed pitch condition [*χ*^2^(2) = 38.563, *p* < 0.001], noise condition [*χ*^2^(1) = 83.730, *p* < 0.001], and the interaction of the two factors [*χ*^2^(2) = 27.885, *p* < 0.001] significantly improved the model. Recognition accuracy did not explain the variance above and beyond the pitch and noise conditions [*χ*^2^(1) = 0.718, *p* > 0.1]. The peak pupil dilation data are also plotted in Fig. 3.

## 4. Discussions

Motivated by the rationale that the dynamic pitch in speech may influence speech perception beyond what can be measured by intelligibility, we collected task-evoked pupil response data to examine the psychophysiological effects of dynamic pitch alteration on speech perception in noise. Consistent with previous findings,^7^ speech recognition accuracy data showed a negative effect of dynamic pitch alteration on speech perception.

Compared to that in the original pitch condition, a weaker pupil response was observed in weakened dynamic pitch condition, while a stronger pupil response was observed in strengthened pitch condition. Stronger pupil responses were demonstrated by faster pupil dilation during the unfolding of speech, and by higher maximum pupil sizes after the speech signal ends. The effect also depended on noise condition and was stronger in the more favorable noise condition (−5 dB SNR). This data provided new information that was not evident from intelligibility data, which would have predicted higher processing effort (and stronger pupil response) for the two pitch altered conditions. A potential explanation for this result is the pupil dilation data in this study also reflect changes in arousal level due to dynamic pitch alteration. Specifically, the strengthened dynamic pitch induced a higher arousal level that was revealed by a stronger pupil response, while the weakened dynamic pitch had an opposite effect. In other words, speech with strengthened dynamic pitch may perceptually be more exciting, while that with weakened pitch may be less exciting, which could also indicate a tired talker. Even the listeners were not asked to discriminate this difference explicitly, the effect of this perceptual difference on pupil response can be involuntary.^11^ This hypothesis is supported by the previous finding showing that larger pitch variation caused a significant increase in skin conductance measure.^10^ This effect of dynamic pitch is potentially due to its role in conveying prosody and emotion^30,31^ and could be modulated by the characteristics of the talker's voice.^32^ In this study, we also found this pitch effect to be stronger in the more favorable noise condition, which is likely due to better audibility and less effortful recognition of speech in noise. To the best of our knowledge, this is the first study using pupillary response to examine the effects of dynamic pitch cues in speech perception in noise. As this work is relatively new, we need more data to further examine this hypothesis. One important question that remains to be answered in a follow-up study is whether/how this hypothesized mechanism interacts with effortful processing of speech in noise and influence pupil response during online speech perception.^13^ Possible experiment designs for investigating this question include using tasks and/or listening conditions that do not require processing effort (e.g., not a recognition task, in quiet) to test the arousal hypothesis. Critically, more realistic speech stimuli should be used in this line of work because the current stimuli of low-context sentences (i.e., IEEE sentences) are potentially more sensitive to processing effort than to arousal effect due to their neutral content. While real-life speech communication contains abundant information through prosodic cues such as dynamic pitch, we need more data to better understand the perceptual effects of these cues, which may not be captured by intelligibility measure alone. For instance, if changes in prosodic cues can increase arousal, it would be of importance to examine the downstream effect of this arousal mechanism in speech communication in real-life scenarios, with more complex speech that lasts for longer duration and has a higher cognitive demand.

Concerning the role of dynamic pitch on speech perception, the finding from the present study serves as a first step in the ongoing work to better understand the paralinguistic functions of this acoustic cue in speech perception. From a theoretical perspective, the results of this study contribute to the literature by shedding light on the complex mechanisms behind pupillometry measure regarding arousal and attention in auditory modality.^14,33–35^ From a clinical perspective, the findings highlight the importance of examining perceptual effects of acoustic cues in speech perception using paradigms and measures beyond sentence intelligibility.
